# Examining the Role of Neuroticism Polygenic Risk in Late Life Cognitive Change: A UK Biobank Study

**DOI:** 10.3390/bs14100876

**Published:** 2024-09-29

**Authors:** Niki Akbarian, Mahbod Ebrahimi, Fernanda C. Dos Santos, Sara Sadat Afjeh, Mohamed Abdelhack, Marcos Sanches, Andreea O. Diaconescu, Tarek K. Rajji, Daniel Felsky, Clement C. Zai, James L. Kennedy

**Affiliations:** 1 Tanenbaum Centre for Pharmacogenetics, Molecular Brain Science, Campbell Family Mental Health Research Institute, Centre for Addiction and Mental Health, Toronto, ON M5T 1R8, Canada; niki.akbarian@mail.utoronto.ca (N.A.); clement.zai@camh.ca (C.C.Z.); 2 Institute of Medical Science, University of Toronto, Toronto, ON M5S 1A8, Canadatarek.rajji@camh.ca (T.K.R.); daniel.felsky@camh.ca (D.F.); 3 Krembil Centre for Neuroinformatics, Centre for Addiction and Mental Health, Toronto, ON M5T 1R8, Canada; 4 Biostatistics Core, Centre for Addiction and Mental Health, Toronto, ON M5T 1R8, Canada; 5 Adult Neurodevelopment and Geriatric Psychiatry Division, Centre for Addiction and Mental Health, Toronto, ON M6J 1H4, Canada; 6 Department of Psychiatry, University of Toronto, Toronto, ON M5T 1R8, Canada; 7 Department of Laboratory Medicine and Pathobiology, University of Toronto, Toronto, ON M5S 1A8, Canada

**Keywords:** cognitive decline, neuroticism, polygenic risk, cognition, personality traits

## Abstract

Cognitive decline is a public health concern affecting about 50 million individuals worldwide. Neuroticism, defined as the trait disposition to experience intense and frequent negative emotions, has been associated with an increased risk of late-life cognitive decline. However, the underlying biological mechanisms of this association remain unknown. This study investigated the relationship between genetic predisposition to neuroticism, computed by polygenic risk score (PRS), and performance in cognitive domains of reasoning, processing speed, visual attention, and memory in individuals over age 60. The sample consisted of UK Biobank participants with genetic and cognitive data available (N = 10,737, 4686 females; mean age = 63.4 ± 2.71). The cognitive domains were assessed at baseline for all participants and seven years later for a subset (N = 645, 262 females; mean age = 62.9 ± 2.44). Neuroticism PRS was not associated cross-sectionally with cognitive measures (*p* > 0.05). However, the trajectory of change for processing speed (β = 0.020; 95% CI = [0.006, 0.035], adjusted *p* = 0.0148), visual attention (β = −0.077; 95% CI = [−0.0985, −0.0553], adjusted *p* = 1.412 × 10^−11^), and memory (β = −0.033; 95% CI = [−0.0535, −0.0131], adjusted *p* = 0.005) was significantly associated with neuroticism PRS. Specifically, a higher genetic predisposition to neuroticism was associated with less decline in these cognitive domains. This trend persisted after sensitivity analysis using complete cases, although it only remained nominally significant for visual attention.

## 1. Introduction

Cognitive decline is characterized as deterioration in cognitive abilities, including reasoning, executive function, sustained attention, and memory, often resulting from impairments in sensory perception and information processing speed [1]. Late-life cognitive impairment presents a pressing public health challenge due to its social and economic burdens caused by loss of functional independence among affected individuals and the associated costs of intensive caregiving [2,3]. Currently, approximately 50 million individuals worldwide live with severe cognitive impairment, a number estimated to increase to 82 million by 2030 and 152 million by 2050 [4].

While some cognitive decline can be a natural aspect of aging [5], it can also be caused by pathologies such as Alzheimer’s disease, Lewy body disease, or cerebrovascular disorders [6]. In addition to age- and health-related conditions, individual differences in the rate of cognitive decline arise from a complex interaction between genetic predispositions, lifestyle factors, demographics, and social determinants [7]. Identifying additional factors influencing the rate of cognitive decline in older adults holds considerable promise for enabling early diagnosis and targeted interventions that can promote greater functional independence and enhance the overall quality of life for older adults.

Recent epidemiological studies have identified neuroticism, a personality trait that is defined as the predisposition to experience intense and frequent negative emotions in response to various sources of stress [8], as a factor that potentially exacerbates the rate of decline in cognition among older adults [9,10,11,12,13]. However, the underlying biological mechanisms associating neuroticism with the risk of late-life cognitive decline remain unknown. One plausible hypothesis posits a shared etiology between neuroticism and risk of cognitive decline, potentially resulting from genetic variants influencing both conditions [14].

Given the polygenic nature of neuroticism [15,16], it may be more effective to assess the association between polygenic liability to neuroticism and cognitive change rather than delving into the role of specific genes and their variants. Several studies have explored the impact of polygenic liability to neuroticism on general cognition, cognitive concerns, or cognitive changes over time [17,18,19,20,21]. However, the findings are inconsistent. Some studies have demonstrated a negative association between polygenic liability to neuroticism and general cognition [18,20], as well as a heightened risk of cognitive impairment [17]. In contrast, other studies have failed to replicate these findings [19,21].

The inconsistency in findings may be due to methodological disparities among previous studies, including variations in participant demographics and ancestries, as well as the choice between longitudinal and cross-sectional study designs. Most studies also featured broad inclusion criteria for age rather than specifically targeting older adults, thus limiting their capacity to assess the influence of genetic predisposition to neuroticism on late-life cognition and cognitive change thoroughly. In addition, some studies examined general cognition, overlooking the heterogeneous nature of cognitive changes across various cognitive domains; that is, while domains such as memory often exhibit more pronounced declines, others, such as language, may not demonstrate comparable deterioration over time [5,22].

Hence, this study aims to overcome these limitations by investigating the relationship between the polygenic risk score (PRS) of neuroticism—a measure estimating an individual’s genetic predisposition to neuroticism based on their genotype profile—and performance of cognitively unimpaired older adults aged 60 years or above in cognitive domains of reasoning, processing speed, visual attention, and memory, as well as changes in these cognitive measures over time, utilizing cross-sectional and longitudinal data from the UK Biobank.

## 2. Materials and Methods

### 2.1. Study Population

Participants in this study were selected from the UK Biobank [23], a prospective cohort study designed to investigate determinants of diseases associated with middle and old age. Between 2006 and 2010 and across 22 centers in Scotland, England, and Wales, UK Biobank recruited around 502,633 participants, aged 40 to 69 years old at the time of recruitment. In 2014, approximately 330,000 participants were reinvited to partake in the UK Biobank imaging study. During this phase, participants completed a series of tests and questionnaires, including assessments of cognitive function. A subsequent follow-up on cognitive tests was conducted in 2021.

Ultimately, all surviving participants, except those who opted out or moved abroad, were invited for the imaging and follow-up assessments; however, data from 330,000 participants were available for the 2014 imaging phase.

For this study, inclusion criteria included age of 60 years or older at baseline (imaging visit), European genetic ancestry, completion of cognitive test battery at the 2014 assessment period, and completion of the Eysenck Personality Questionnaire—Revised Short Form for measuring neuroticism. Exclusion criteria encompassed the diagnosis of any type of dementia or neurodegenerative diseases impacting cognitive abilities, as well as a history of mood disorders (i.e., manic episode, bipolar affective disorder, depressive episode, recurrent depressive episode, persistent mood disorders, or other/unspecified mood disorders), and mental and behavioral disorders due to psychoactive substance abuse, schizophrenia, schizotypal, or delusional disorders.

### 2.2. Measures

Neuroticism: The 12-item neuroticism scale of the Eysenck Personality Questionnaire—Revised Short Form (EPQ-R) was administered to assess neuroticism [24]. The EPQ-R has been validated to measure neuroticism in older individuals as it has shown strong correlation with the neuroticism domain of the NEO Five-Factor Inventory (r = 0.85) [25]. In the EPQ-R questionnaire, participants were presented with 12 questions evaluating traits such as nervousness, loneliness, irritability, mood swings, guilt, feeling fed up, being a worrier, and experiencing hurt feelings. Response options included “Yes”, coded as 1, “No”, coded as 0, and “Do not know” or “Prefer not to answer”, both coded as missing. Therefore, a participant could achieve a total score of 12 on the neuroticism scale if they responded affirmatively to all questions.

Cognitive Measures: Four cognitive domains of reasoning, processing speed, visual attention, and memory were assessed. The administered cognitive test battery was uniquely designed for the UK Biobank, allowing for unsupervised test administration. Although this unique design resulted in non-standardized tests, it has been shown that the UK Biobank cognitive tests demonstrate substantial test-retest reliability, and they correlate moderately to strongly with well-validated cognitive tests assessing the same cognitive domains [26]. Specifically, assessments for reasoning and processing speed exhibited test-retest correlations exceeding 0.5, and tests for memory and visual attention displayed moderate correlations, ranging between 0.4 and 0.5 [26].

(1) Reasoning: Reasoning or fluid intelligence refers to logic and reasoning abilities that are distinct from acquired knowledge and are crucial for problem-solving. In the assessment, participants were presented with 13 multiple-choice questions and were instructed to complete as many questions as possible within a two-minute timeframe. The questions covered numeric addition (e.g., adding the numbers 1, 2, 3, 4, 5), word interpolation (e.g., Bud is to flower as child is to?), arithmetic sequence recognition (e.g., 150… 137… 125… 114… 104… What comes next?), and subset inclusion logic (e.g., If some flinks are plinks and some plinks are stinks, then some flinks are definitely stinks?). Subsequently, fluid intelligence was measured by totaling the number of questions answered correctly.
(2) Processing Speed: The symbol digit substitution test was employed as a web-based questionnaire to assess processing speed [27]. During the test, participants were allotted approximately three minutes to match symbols in a series of grids to corresponding numbers based on a provided key. The processing speed score was determined by the number of matches made correctly.
(3) Visual Attention: The trail making test (part A), implemented as a web-based questionnaire in the UK Biobank, was employed to assess visual attention. In this test, participants were asked to draw lines to connect circled numbers in a numerical sequence (e.g., 1–2–3). The score was derived from the time in seconds taken to accurately connect all the circles, with a higher time indicating poorer performance. For some participants, a value of 0 was recorded as the time taken to complete the test. These participants were excluded from the analysis as a time 0 indicated that they did not complete the test.
(4) Memory: Episodic memory was evaluated using the pairs matching test. During the assessment, participants were tasked with memorizing the positions of as many matching pairs of cards as possible. Subsequently, the cards were turned face down, and participants were required to identify as many pairs as they could with the fewest attempts. The test consisted of two or three rounds: the first round with 3 pairs of cards, the second with 6 pairs, and the third with 8 pairs. Participants progressed to the third round only if they made 0 errors or 1 error in the second round. The score for this test was determined by the number of incorrect matches in a round, where a higher score indicated poorer performance.

The data for all the cognitive tests used for analysis were collected throughout the first imaging visit (30 October 2014 to 21 April 2015) and the follow-up visit (10 February 2021 to 11 January 2022).

For further information about the dataset and cognitive tests, please refer to UK Biobank showcase website at https://biobank.ndph.ox.ac.uk/showcase/ (accessed on 1 August 2024).

### 2.3. DNA Collection, Genotyping and Genetic Quality Control

UK Biobank collected genomic DNA from the saliva and blood samples of participants. Genotyping, performed by Affymetrix (now part of ThermoFisher Scientific, Waltham, MA, USA), utilized two purpose-designed arrays: the UK BiLEVE Axiom array for 50,000 participants and the UK Biobank Axiom array for the remaining 450,000. In total, there were 805,426 markers in GRCh37 coordinates in the genotype data.

The Wellcome Trust Centre for Human Genetics (WTCHG) at Oxford University, the Krembil Centre for Neuroinformatics (KCNI) at CAMH, and our group at The Tanenbaum Centre for Phatmacogenetics at CAMH performed the genetic quality control (QC) procedures. Throughout the initial QC executed by WTCHG, samples with sex aneuploidy, non-European ancestry, and non-inclusion in the principal components analysis (PCA) were excluded. In addition, SNPs with insertion/deletion polymorphisms, call rate < 95%, deviation from Hardy–Weinberg equilibrium (*p* < 1 × 10^−10^), minor allele frequency < 0.01%, or an imputed information (INFO) score < 0.8 were also removed.

For the purpose of this study, genetic data for individuals meeting the inclusion criteria were extracted (N = 10,737). For further QC, the GWAS analysis toolkit of PLINK v1.9 [28] and RStudio v4.3.3 software [29] were utilized. Individuals with excessive heterozygosity (>three standard deviations from the sample mean), call rate < 95%, or high relatedness (pi-hat > 0.2) were excluded. Additionally, SNPs with minor allele frequency < 1%, missing call rate < 99%, or not in Hardy–Weinberg equilibrium (*p* < 1 × 10^−6^) were removed.

### 2.4. Summary Statistics Quality Control

To calculate the PRS for neuroticism, publicly available summary statistics released by the Genetics of Personality Consortium was utilized [15]. The summary statistics consisted of a meta-analysis of GWAS on neuroticism, incorporating data from 63,661 participants across 29 discovery cohorts and 9786 participants in a replication cohort. The participants included in the cohorts came from Europe, the United States, or Australia, all with European genetic ancestry. No data from the UK Biobank were included in the GWAS.

### 2.5. Polygenic Score Calculation

The PRS of neuroticism was derived from clumping and thresholding (C + T) performed by PRSice2 software v2.3.5 [30].

For clumping, variants with the lowest *p*-value in the discovery GWAS, referred to as the index SNP, were selected. Subsequently, within a genetic distance of 250 kb from the index SNP, only the variants that were weakly correlated (r^2^ = 0.1) with the index SNP were retained to prune redundant correlated effect. For thresholding, 10000 permutations were conducted—with default values for the starting *p*-value threshold (i.e., 5 × 10^−8^) and the step size of the threshold (i.e., 5 × 10^−5^)—to choose the *p*-value threshold at which SNPs could explain the greatest amount of variance in the neuroticism phenotype. Subsequently, linear regression models were constructed, with neuroticism scores from EPQ-R as the dependent variable, representing the phenotype, and neuroticism PRS at different *p*-value thresholds as independent variables. R2 was used to calculate the proportion of variance in the neuroticism phenotype explained by the derived PRS.

The PRS constructed at the *p*-value threshold of 0.1231 explained the highest amount of variance in the neuroticism phenotype in the target sample. Consequently, this PRS was utilized for the subsequent analyses.

### 2.6. Statistical Analysis

To assess the cross-sectional association between neuroticism PRS and cognitive measures, linear regression models were constructed for each cognitive domain, using the values at baseline. Age, sex, and years of education were added to the models as covariates. Since population structure may be a principal source of confounding in GWAS and PRS analysis [31], the first 10 principal components (PCs) were also included as covariates to account for population stratification.

For the longitudinal analyses, linear mixed effect models were computed to assess the relationship between the PRS of neuroticism and changes in tested cognitive domains from baseline to follow-up. For each cognitive test, a separate mixed effect model was constructed using the “lmer” function in the “lme4” [32] and “lmerTest” [33] packages in RStudio v4.3.3 [29]. Since lme4 can handle missing data through pairwise deletion and the maximum likelihood estimate [32], all participants with at least one valid cognitive measure were included in the primary analysis. In each model, cognitive scores at baseline and follow-up were set as the outcome. Moreover, neuroticism PRS and time (baseline and follow-up), as well as their interaction, were included as predictors. Age, sex, baseline cognition, as well as the first 10 PCs, were added to the models as covariates. The IDs of participants and the study site (i.e., Cheadle, Newcastle, Reading, or Bristol) were set as the random intercepts. To assess the significance of the association between neuroticism PRS and changes in cognitive measures over time, we specifically examined the significance of the interaction between neuroticism PRS and time. The estimated marginal means (EMMs) were subsequently used to visualize and interpret estimated trajectories at different values (i.e., 25th, 50th, 75th percentiles) of neuroticism PRS. *p*-values were adjusted for multiple testing using the Benjamini–Hochberg method [34], with the significance threshold set to corrected *p* < 0.05.

### 2.7. Sensitivity Analysis

**Figure 1:**
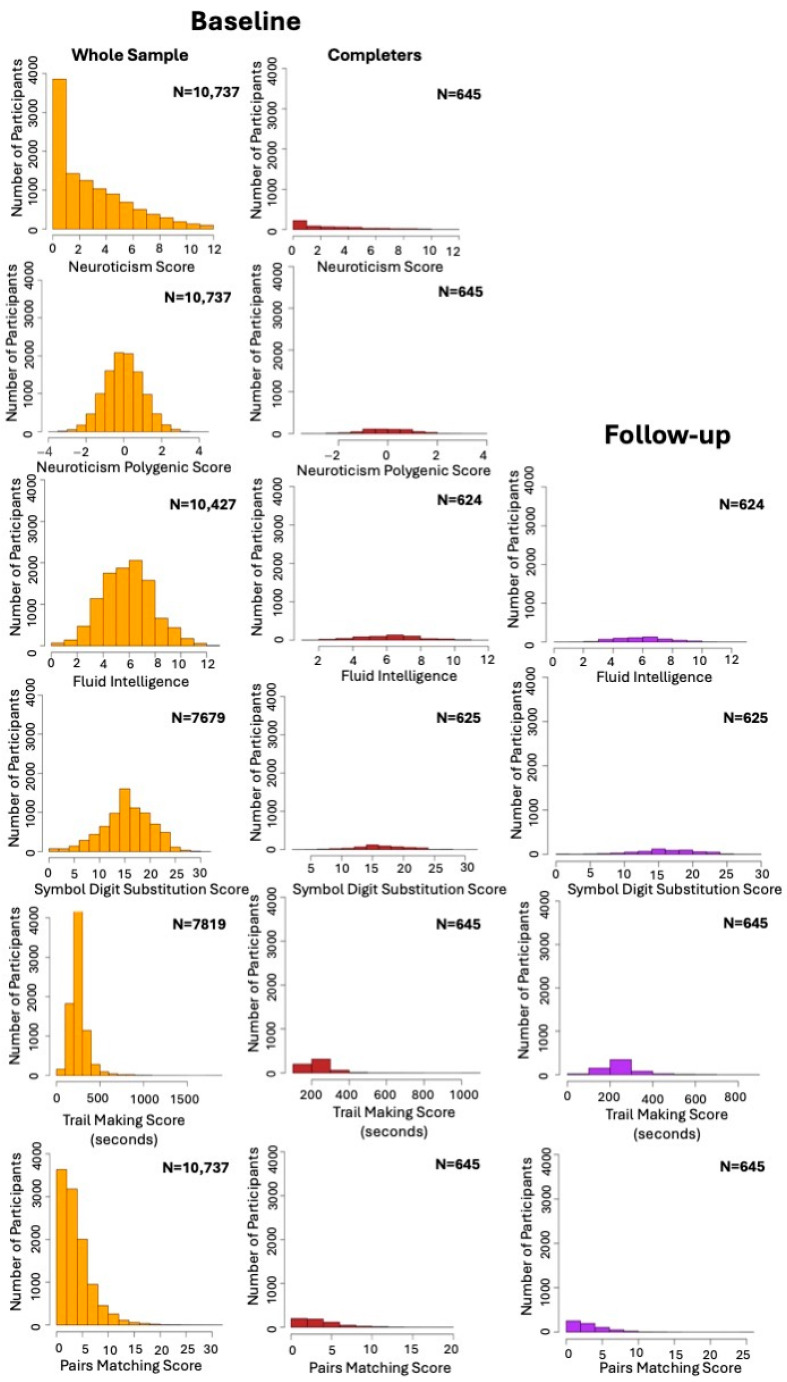
Distribution of variables at baseline and follow-up. The histograms in orange represent distribution of variables for the whole sample at baseline. Histograms in red represent distribution of variables at baseline for participants who had complete longitudinal data. Histograms in purple represent distribution of variables at follow-up assessment for participants who had complete data. N = sample size.

Due to the skewed distribution of the variables (Figure 1), the longitudinal analysis was conducted again for cognitive measures, using the log-transformed values of variables. In addition, for sensitivity analysis, the same statistical analysis was conducted once again, this time solely with participants who had both baseline and follow-up data to evaluate the effect of missing data.

## 3. Results

### 3.1. Demographic Characteristics

The demographic characteristics of participants at baseline and follow-up are summarized in Table 1. In total, 10,737 individuals (4686 females) with a mean age of 63.4 years (SD = 2.71) were included in the baseline assessment. A subset of 645 of these participants (262 females) completed the follow-up assessment as well. As some participants did not complete the cognitive battery in its entirety, the number of participants varied across tests. The missing rate for each cognitive test at baseline and follow-up, as well as the demographic characteristics of completers and participants lost to follow-up, are reported in Table 1.

### 3.2. Cross-Sectional Analysis

In the total sample at baseline, the PRS of neuroticism was not significantly associated with any of the cognitive measures, including fluid intelligence (β = 0.0237; 95% CI = [−0.0153, 0.0628], *p* = 0.2335, N = 10427), symbol digit substitution (β = −0.0199; 95% CI = [−0.1289, 0.0892], *p* = 0.7211, N = 7679), trail making (β = 0.9644; 95% CI = [−1.5115, 3.4405], *p* = 0.4452, N = 7819), or pairs matching (β = 0.0466; 95% CI = [−0.1732, 0.2664], *p* = 0.6773, N = 10737).

### 3.3. Longitudinal Analysis

In the mixed effect models, the interaction between neuroticism PRS and time was statistically significant for trail making (β = −0.077; 95% CI = [−0.0985, −0.0553], adjusted *p* = 1.41 × 10^−11^) and pairs matching scores (β = −0.033; 95% CI = [−0.0535, −0.0131], adjusted *p* = 0.003), and nominally significant for symbol digit substitution (β = 0.020; 95% CI = [0.0006, 0.0405], unadjusted *p* = 0.050). No significant interaction was found for fluid intelligence (β = 0.014; 95% CI = [−0.0019, 0.0301], adjusted *p* = 0.0862), as shown in Table 2.

To further interpret these interactions, EMMs were used to assess the trajectory of change for each cognitive measure across three percentiles (75th, 50th, and 25th) of neuroticism PRS.

#### 3.3.1. Trail Making

**Figure 2:**
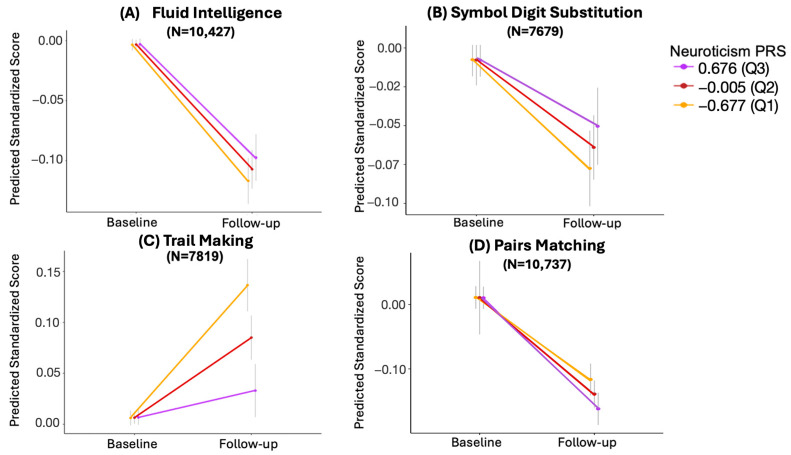
Cognitive performance trajectories across different percentiles of neuroticism PRS for (**A**) fluid intelligence, (**B**) symbol digit substitution, (**C**) trail making, and (**D**) pairs matching. The y-axis represents the scores predicted by models that include the interaction term between neuroticism PRS and time, as well as the covariates of sex, age, baseline cognitive score, and the first 10 genetic principal components. Neuroticism PRS is divided by percentiles, with purple, red, and yellow representing the 75th (Q3), 50th (Q2), and 25th (Q1) percentiles, respectively. The grey vertical lines represent 95% confidence intervals. N represents the sample size.

As illustrated in Figure 2C, at follow-up, trail making completion time was notably lower in the 75th percentile of neuroticism PRS compared to the 50th and 25th percentiles. Suggestively, a higher PRS for neuroticism was associated with a significantly lower increase in the completion time for the trail making test from baseline to follow-up (Figure 2C).

#### 3.3.2. Pairs Matching

For pairs matching, the number of incorrect matches depicted a higher increase in the 25th percentile of neuroticism PRS at follow-up compared to the 50th and 75th percentiles; that is, a higher PRS of neuroticism was associated with less decline in performance for the pairs matching test, consistent with the findings for trail making (Figure 2D).

#### 3.3.3. Symbol Digit Substitution

A similar trend to trail making and pairs matching was observed for the symbol digit substitution scores, as displayed in Figure 2B. In particular, symbol digit substitution scores exhibited a smaller decline in the 75th percentile of neuroticism PRS compared to the 50th and 25th percentiles. This suggests that a higher neuroticism PRS was nominally associated with a smaller decline in symbol digit substitution scores from baseline to follow-up.

#### 3.3.4. Fluid Intelligence

Although not reaching statistical significance (*p* > 0.05), a similar trend of association between higher neuroticism PRS and lesser decline was observed for fluid intelligence scores (Figure 2A).

### 3.4. Longitudinal Analysis with Log-Transformed Values

**Figure 3:**
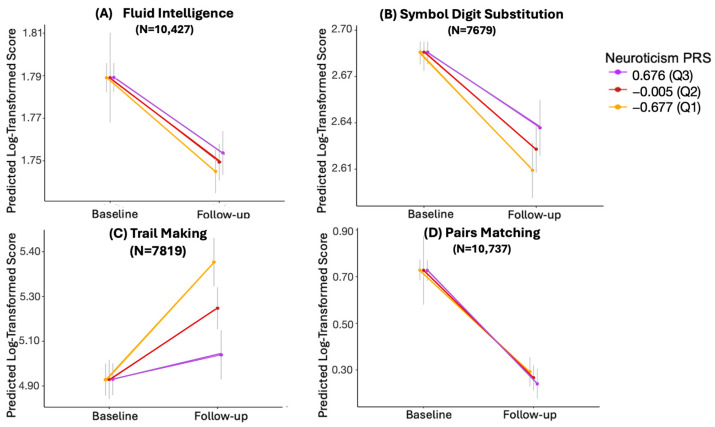
Cognitive performance trajectories across different percentiles of neuroticism PRS for (**A**) fluid intelligence, (**B**) symbol digit substitution, (**C**) trail making, and (**D**) pairs matching with log-transformed values. The y-axis represents the log-transformed scores predicted by models that include the interaction term between neuroticism PRS and time, as well as the covariates of sex, age, baseline cognitive score, and the first 10 genetic principal components. Neuroticism PRS is divided by percentiles, with purple, red, and yellow representing the 75th (Q3), 50th (Q2), and 25th (Q1) percentiles, respectively. The grey vertical lines represent 95% confidence intervals. N represents the sample size.

Given the skewed distribution of values for the cognitive tests, mixed-effect models were reconstructed using log-transformed values of cognitive measures. As depicted in Figure 3, the trends observed were similar to those from the previous analysis; that is, a higher PRS of neuroticism was associated with a lower decline in scores of fluid intelligence, symbol digit substitution, trail making, and pairs matching. However, following adjustment for multiple testing, the interaction between neuroticism PRS and time was only statistically significant for the symbol digit substitution (β = 0.020; 95% CI = [0.0055, 0.0352], adjusted *p* = 0.0148) and trail making test (β = −0.159; 95% CI = [−0.2130, −0.1040], adjusted *p* = 5.04 × 10^−8^), illustrated in Table 3.

### 3.5. The Effect of Missing Longitudinal Data

**Figure 4:**
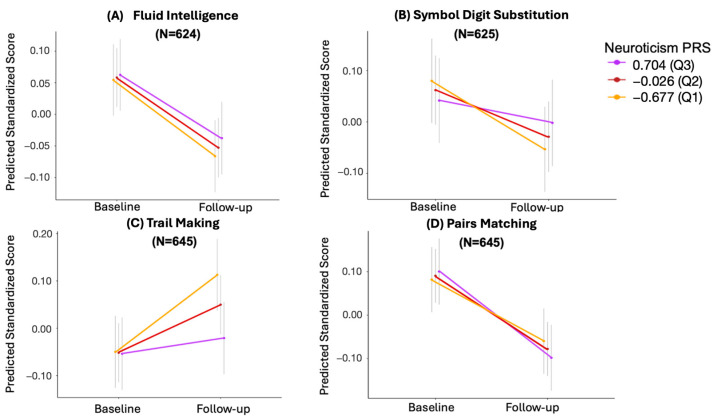
Cognitive performance trajectories across different percentiles of neuroticism PRS in participants that had both baseline and follow-up data for (**A**) fluid intelligence, (**B**) symbol digit substitution, (**C**) trail making, and (**D**) pairs matching. The y-axis represents the scores predicted by models that include the interaction term between neuroticism PRS and time, as well as the covariates of sex, age, baseline cognitive score, and the first 10 genetic principal components. Neuroticism PRS is divided by percentiles, with purple, red, and yellow representing the 75th (Q3), 50th (Q2), and 25th (Q1) percentiles, respectively. The grey vertical lines represent 95% confidence intervals. N represents the sample size.

The analysis described thus far was based on participants who had complete data at baseline, regardless of whether they completed follow-up assessment. To further investigate the impact of missing follow-up data, the aforementioned analysis was repeated on the subset of participants with both baseline and follow-up data available (N = 645). Consistently, we observed a trend wherein a higher PRS of neuroticism was associated with less decline in measures of fluid intelligence, symbol digit substitution, trail making, and pairs matching (Figure 4). However, nominal significance was only attained for the trail making test (β = −0.094; 95% CI = [−0.1818, −0.0058], unadjusted *p* = 0.038, adjusted *p* = 0.152), as depicted in Table 4.

## 4. Discussion

Recent epidemiological studies have demonstrated an association between neuroticism and late-life cognitive decline [9,10,11,12,13]. However, there is still a gap in the literature regarding the genetic basis of this relationship. Our results suggest that a higher genetic predisposition to neuroticism may be associated with a lower decline in cognitive function, particularly in attentional abilities.

These findings present a novel perspective on the genetic relationship between neuroticism and late-life cognitive changes. In contrast to previous studies that either reported a negative association or no relationship [17,18,19,20,21], we found a positive genetic association between neuroticism and cognitive decline. Methodological differences may explain these discrepancies in findings. For instance, some of the previous studies were not specific to older adults and had a cross-sectional design. In addition, prior research often assessed changes in general cognition rather than separate domains of cognition, which are shown to be changed differently with age [5,22]. Moreover, the inclusion and exclusion criteria, as well as covariates considered in the analyses, were different among studies, potentially giving rise to contradictory findings. For instance, several studies did not control for a history of mood and mental disorders.

Furthermore, despite neuroticism often being viewed as a negative trait associated with poor health outcomes, including heightened risks of common mental and cardiovascular disorders [35,36], emerging research suggests a more nuanced relationship between neuroticism and health outcomes. Depending on various circumstances, neuroticism can exert either positive or negative impacts on health [37,38]. For instance, a study of 321,456 people from the UK Biobank revealed that higher levels of neuroticism were associated with an 8% reduction in all-cause mortality (hazard ratio = 0.92; 95% CI = [0.89, 0.95]) after adjusting for covariates, including age, sex, health behaviors (e.g., smoking status, frequency of alcohol intake, etc.), physical attributes (e.g., body mass index, systolic blood pressure, etc.), existing illness (diagnosis of vascular or heart problems, diabetes, etc.), and self-rated health [39]. Indeed, evidence suggests that neuroticism, considered independently of other influential factors such as health behaviors and comorbidities that can exacerbate health conditions, may have a positive impact on health outcomes, as individuals with higher levels of neuroticism tend to be more vigilant about their health [39,40], which leads them to seek medical attention and utilize healthcare services more readily [40,41]. In our sample, all participants were cognitively unimpaired and free from mood disorders, eliminating the potential role of variables and conditions that could influence the association between neuroticism and late-life cognitive change. Moreover, our study specifically examined the genetics of neuroticism, which remain relatively unaffected by external variables, allowing us to examine the potential direct impact of neuroticism on late-life cognitive change, independent of comorbidities or other health factors.

Moreover, our results suggest that a genetic predisposition to neuroticism is differentially associated with trajectory of change in various domains of cognition. Particularly, the genetic liability to neuroticism was negatively associated with decline in attention, processing speed, and memory, but not reasoning. This is partially in line with previous research showing that neuroticism is positively associated with attentional investment [42] and overall information processing speed, although at the expense of accuracy [43], but not overall reasoning abilities in older adults [44]. In addition, it has been suggested that individuals with higher neuroticism tend to have higher cerebral blood flow velocity during cognitive tasks, indicating greater expenditure of mental effort, which, in turn, results in heightened vigilance and sustained task focus, despite faster depletion of cognitive resources [45]. These compensatory mechanisms might help explain how genetic predisposition to neuroticism may preserve attentional abilities in older adults, counteracting some of the typical age-related decline in attention.

This research had several strengths, including a relatively large sample of older adults, longitudinal data, and comprehensive tasks that measured four different domains of cognition. However, the results of our study should be viewed in the light of its limitations. First, as described in results, a substantial proportion of participants who completed the baseline assessment did not have longitudinal data available, despite invitations to complete follow-up assessments [46]. Nevertheless, observing the consistent trend of results following sensitivity analysis mitigated the potential impact of this limitation on the findings. Another limitation is that the follow-up assessment occurred partially during the COVID-19 pandemic, which may have influenced participation and introduced selection bias, as only certain individuals were able or chose to participate during this time. Furthermore, to avoid data overlap between the summary statistics and the target data (i.e., UK Biobank) used for calculating the PRS, the most recent GWAS for neuroticism [16] was not used as it included UK Biobank participants. In addition, while more advanced techniques, such as Bayesian shrinkage [47] or penalized regression modelling [48], are now recommended for calculating PRS for better predictive power, our study utilized clumping and thresholding with PRSice2 software, due to computational constraints and the structure of the summary statistics used. Last, the distribution of scores for several tests, including pairs matching, was skewed in our sample, raising the possibility that ceiling effects may have limited the detection of changes over time, ultimately affecting the results. Despite these limitations, this research represents a significant step forward in understanding the relationship between polygenic predisposition to neuroticism and cognitive change in older adults.

## 5. Conclusions

Overall, this study investigated the genetic relationship between neuroticism and late-life cognitive change. While the observed effect sizes were small, our results suggest that polygenic predisposition to neuroticism may be associated with less decline in attention in older adults. Further research is required to better understand the underlying mechanisms driving the relationship between neuroticism and cognitive decline in older adults.

## Figures and Tables

**Table 1:** Demographic characteristics and average cognitive test scores of participants at baseline and follow-up. Completers are participants with data at both time points.

Demographic Characteristics at Baseline							
				Completers (N = 645)	Lost to Follow Up (N = 10,092)		Overall (N = 10,737)
Sex							
			Female	262 (40.6%)	4424 (43.8%)		4686 (43.6%)
			Male	383 (59.4%)	5668 (56.2%)		6051 (56.4%)
Age (Years)							
			Mean (SD)	62.9 (2.44)	63.5 (2.72)		63.4 (2.71)
Neuroticism							
			Mean (SD)	3.26 (2.88)	3.20 (2.92)		3.21 (2.91)
Neuroticism PRS							
			Median [Min, Max]	−0.0261 [−3.10, 3.71]	−0.0047 [−3.51, 4.02]		−0.00486 [−3.50, 4.01]
**Cognitive Scores**							
**Baseline**					**Follow-up**		
		Completers (N = 645)	Lost to Follow Up (N = 10,092)	Overall (N = 10,737)			Completers (N = 645)
Fluid Intelligence					Fluid Intelligence		
	Mean (SD)	6.71 (1.97)	6.35 (1.99)	6.37 (2.00)		Mean (SD)	6.47 (1.89)
	Missing	8 (1.2%)	302 (3.0%)	310 (2.9%)		Missing	21 (3.1%)
Symbol Digit Substitution					Symbol Digit Substitution		
	Mean (SD)	17.7 (4.08)	16.8 (4.44)	16.0 (4.85)		Mean (SD)	16.4 (4.95)
	Missing	44 (6.8%)	3014 (29.9%)	3058 (28.5%)		Missing	20 (3.1%)
Trail Making					Trail Making		
	Mean (SD)	238 (88.4)	259 (114)	258 (113)		Mean (SD)	250 (101)
	Missing	35 (5.4%)	2883 (28.6%)	2918 (27.2%)			
Pairs Matching					Pairs Matching		
	Mean (SD)	4.10 (2.90)	4.18 (3.27)	4.18 (3.25)		Mean (SD)	3.61 (2.79)

N = sample size; SD = standard deviation.

**Table 2:** Summary of results for the interaction between neuroticism PRS and time in mixed effects models for each cognitive test. The models included sex, age, baseline cognitive scores, and the first 10 genetic principal components as covariates. The *p*-values were adjusted using the Benjamini–Hochberg method, with a significance threshold of 0.05.

	Estimate	95% CI	SE	t	*p*-Value	Adjusted *p*-Value	N
Fluid Intelligence	0.014	−0.0019, 0.0301	8.174 × 10^−3^	1.715	0.086	0.086	10427
Symbol Digit Substitution	0.020	0.0006, 0.0405	1.033 × 10^−2^	1.964	**0.050**	0.066	7679
Trail Making	−0.077	−0.0985, −0.0553	1.104 × 10^−2^	−6.966	**3.53 × 10^−12^**	**1.41 × 10^−11^**	7819
Pairs Matching	−0.033	−0.0535, −0.0131	1.032 × 10^−2^	−3.229	**0.001**	**0.003**	10737

95% CI = 95% confidence interval; SE = standard error; N = sample size. The bold values represent significant *p*-values with a set threshold of 0.05.

**Table 3:** Summary of results for the interaction between neuroticism PRS and time in mixed-effects models for each cognitive test with log-transformed values. The models included sex, age, log-transformed baseline cognitive score, and the first 10 genetic principal components as covariates. The *p*-values were adjusted using the Benjamini–Hochberg method, with a significance threshold of 0.05.

	Estimate	95% CI	SE	t	*p*-Value	Adjusted *p*-Value	N
Fluid Intelligence	0.006	−0.0020, 0.0147	4.277 × 10^−3^	1.483	0.138	0.152	10,427
Symbol Digit Substitution	0.020	0.0055, 0.0352	7.585 × 10^−3^	2.679	**0.007**	**0.0148**	7679
Trail Making	−0.159	−0.2130, −0.1040	2.782 × 10^−2^	−5.698	**1.26 × 10^−8^**	**5.04 × 10^−8^**	7819
Pairs Matching	−0.038	−0.0892, 0.0138	2.631 × 10^−2^	−1.433	0.152	0.152	10,737

95% CI = 95% confidence interval; SE = standard error; N = sample size. The bold values represent significant *p*-values with a set threshold of 0.05.

**Table 4:** Summary of results for the interaction between neuroticism PRS and rime in mixed-effects models for each cognitive test for participants with complete data only. The models included sex, age, baseline cognitive score, and the first 10 genetic principal components as covariates. The *p*-values were adjusted using the Benjamini–Hochberg method, with a significance threshold of 0.05.

	Estimate	95% CI	SE	t	*p*	Adjusted *p*-Value	N
Fluid Intelligence	0.015	−0.0509 0.0803	3.365 × 10^−2^	0.434	0.665	0.665	624
Symbol Digit Substitution	0.021	−0.0530, 0.0954	3.812 × 10^−2^	0.557	0.578	0.665	625
Trail Making	−0.094	−0.1818, −0.0058	4.514 × 10^−2^	−2.079	**0.038**	0.152	645
Pairs Matching	−0.041	−0.1277, 0.0455	4.444 × 10^−2^	−0.926	0.354	0.665	645

95% CI = 95% confidence interval; SE = standard error; N = sample size. The bold values represent significant *p*-values with a set threshold of 0.05.

## Data Availability

Restrictions apply to the availability of data used in this study. Data were obtained from the UK Biobank (Project ID: 61530) and are available upon application submission to the UK Biobank.
